# The General Medical Council

**Published:** 1899-12-09

**Authors:** 


### The General Medical Council.

The sixty eighth session of the General Medical Council, which was opened on Tuesday, November 28th, and concluded on the following Tuesday, December 5th, has not been particularly eventful. Its commencement was enlivened by a sharp encounter between Mr. Brudenell Carter and Mr. Horsley, the latter of whom had published in the Manchester *Medical Guild Quarterly* of October 1st, and had uttered at a meeting at Newcastle of later date, certain statements derogatory to the Society of Apothecaries, declaring them, among other things, to be a non-medical body, who "knew no more of medicine than they did of bootmaking," and who "had received no medical education." Mr. Carter showed that only duly qualified medical practitioners, since 1815, have been eligible for election into the court or governing body of the society, his remarks being received with applause by many members of the Council, who then heard Mr. Horsley in reply. At the conclusion of this episode, the next business was to ask the president to nominate a deputation to the President of the Local Government Board, charged with the duty of impressing upon him the great importance of an amendment of the present registration law, under which registrars are permitted to receive what is called "information" as to the causes of death from non-medical and other unqualified persons, and which affords great facilities for the murder of children and even of adults. The President afterwards announced that the deputation would consist, in addition to himself, of Sir Dyce Duckworth, Dr. Glover, and Mr. Carter. A report was read from the Executive Committee, on the applications of several persons who had been removed from the register for various offences, and who made application to be restored. The committee recommended that the application should be granted in the cases of Robert Masters Theobald, and of George Hamilton Wyse, that it should be refused in the cases of Robert Alexander Douglas Lithgow and of William John Patterson, and in the case of Samuel Frederic Murphy they made no recommendation. The several recommendations were approved by the Council, which then passed on to the consideration of a motion by Mr. Brown, which had for its object to restrain the Obstetrical Society of London and other bodies and individuals from granting to midwives certificates of fitness to attend cases of natural labour. Over this futile and foolish business nearly two days were wasted with no result, and the rest of the second day was equally wasted in another discussion started by Mr. Brown, who wished the Council to pass resolutions recommending the several licensing corporations of each division of the United Kingdom to combine for the purpose of establishing in each a "one-portal" system of examination. The Council has, however, no power to give effect to such a recommendation. On Thursday the Council considered a charge which had been preferred against Dr. William Stewart, and adjourned from the last session, of having covered an unqualified person. Evidence was given that Dr. Stewart had abandoned the practices complained of, and the Council determined not to proceed further in the case. A similar adjourned hearing took place of a charge against a dentist, Mr. Clement Maguire Kershaw, and the same course was pursued. The name of Henry Louis Goodman, who had been convicted of misdemeanour, was ordered to be erased from the Dentists' Register; and the Council then proceeded to hear a charge of covering which was brought against Richard Thomas Williams, of Cwmavon, Glamorganshire, which occupied a large part of the second day, and was finally adjudged not to have been proved to the satisfaction of the Council. William Matthews Joyce, of Birmingham, who had been convicted at Warwick of having been drunk and disorderly, and, on a subsequent occasion, of assault, appeared before the Council, and was ordered to attend again in six months, and to be provided with evidence of good behaviour; and the name of Frederic William Kirkham, who had been convicted of fraud, was ordered to be erased from the Medical Register.

On Saturday there was some rather important business, arising out of the prosecution of the late Mr. Hunter, a Licentiate of the Society of Apothecaries, for calling himself "physician"; and a letter was read from Mr. Upton, the clerk to the Society, impugning the accuracy of the judgment, and asking the Council to co-operate with the Society in obtaining a final decision of the question at issue. The consideration of this proposal was referred by the Council to a special committee, to be named by the President, who afterwards mentioned Mr. Bryant, Dr. Pye-Smith, and Dr. MacAlister as his coadjutors. A report embodying Mr. Muir Mackenzie's opinion as to the laws regulating medical practice in the United Kingdom, with special reference to what may be done in this country by foreign practitioners, was presented and discussed; and a resolution was passed inviting the Government to consider the question in relation to the privileges afforded to English practitioners in Italy, and to approach the Italian Government with a view to the establishment of reciprocity between the two countries. The next business was the consideration of the regulations of the Conjoint Board of England, under which, it was maintained, the first five years of what was intended by the Council to be medical study was spent in possibly deceptive or inefficient science teaching in schools; and a strong opinion was expressed that the regulations in question should be altered, and brought into harmony with the intentions of the Council. Finally, the President was requested to confer with the authorities of the Conjoint Board upon the question.

Perhaps the most important business of the session, a report from the Education Committee upon the possibility of definitely raising the standard of preliminary education for the profession, which, with many other questions, had been pushed aside, did not come on for discussion until Monday, December 4th, and was not finally dealt with until the last day of the session. The Education Committee had obtained the assistance of distinguished educational experts, namely: For England, the Rev. T. W. Sharpe, C.B., formerly Her Majesty's Senior Inspector of Schools; for Scotland, Professor W. S. M'Cormick, LL.D., of the University of St. Andrews; and for Ireland, Joseph McGrath, LL.D., one of the secretaries of the Royal University. After a very careful inquiry, and after consultation with these gentlemen, the committee were not able to speak hopefully as to the prospect of raising the present standard of preliminary education, otherwise than very gradually; and their report was finally referred back by the Council in order that the views of the experts, on which it was to some extent founded, might be set forth at greater length and in a more explicit manner. The remaining business was mostly formal, and consisted of receiving the reports of various committees. Among these that of the Pharmacopœia Committee deserves notice, in that it recorded that the whole of the expenses of the preparation and publication of the last issue had been covered, and that the committee had in hand a balance of more than £1,100. An addendum for the Colonies and India is in active preparation, and will probably be published early in the coming year. Reports which required discussion were mostly deferred until the next session of the Council, and a hearty vote of thanks to the President brought the proceedings to a close.

